# Exosomes as a delivery tool of exercise-induced beneficial factors for the prevention and treatment of cardiovascular disease: a systematic review and meta-analysis

**DOI:** 10.3389/fphys.2023.1190095

**Published:** 2023-09-29

**Authors:** Zhijie Lai, Jiling Liang, Jingfeng Zhang, Yuheng Mao, Xinguang Zheng, Xiang Shen, Wentao Lin, Guoqin Xu

**Affiliations:** ^1^ Department of School of Physical Education, Guangzhou College of Commerce, Guangzhou, China; ^2^ College of Sports Medicine, Wuhan Sports University, Wuhan, China; ^3^ College of Humanities Education, Foshan University, Foshan, China; ^4^ Department of Sports and Health, Guangzhou Sport University, Guangzhou, China; ^5^ Department of School of Physical Education, Zhuhai College of Science and Techology, Zhuhai, China; ^6^ Guangdong Provincial Key Laboratory of Physical Activity and Health Promotion, Guangzhou Sport University, Guangzhou, China

**Keywords:** exercise, exosomes, extracellular vesicles, cardiovascular disease, meta-analysis

## Abstract

Exercise-derived exosomes have been identified as novel players in mediating cell-to-cell communication in the beneficial effects of improving cardiovascular disease (CVD). This review aimed to systematically investigate exosomes as delivery tools for the benefits of exercise in the prevention and treatment of CVD and summarize these outcomes with an overview of their therapeutic implications. Among the 1417 articles obtained in nine database searches (PubMed, EBSCO, Embase, Web of Science, CENTRAL, Ovid, Science Direct, Scopus, and Wiley), 12 articles were included based on eligibility criteria. The results indicate that exercise increases the release of exosomes, increasing exosomal markers (TSG101, CD63, and CD81) and exosome-carried miRNAs (miR-125b-5p, miR-122-5p, miR-342-5p, miR-126, miR-130a, miR-138-5p, and miR-455). These miRNAs mainly regulate the expression of MAPK, NF-kB, VEGF, and Caspase to protect the cardiovascular system. Moreover, the outcome indicators of myocardial apoptosis and myocardial infarction volume are significantly reduced following exercise-induced exosome release, and angiogenesis, microvessel density and left ventricular ejection fraction are significantly increased, as well as alleviating myocardial fibrosis following exercise-induced exosome release. Collectively, these results further confirm that exercise-derived exosomes have a beneficial role in potentially preventing and treating CVD and support the use of exercise-derived exosomes in clinical settings.

## Introduction

Cardiovascular disease (CVD) is an essential reason for the increase in the global incidence rate and mortality and poses a severe threat to human health. Physical inactivity is the leading risk factor for CVD. The beneficial role of regular physical exercise in the prevention and treatment of CVD has long been appreciated (Mann and Rosenzweig, 2012). Exercise training is considered safe, simple, and has extensive health effects (Powers et al., 2014). It can effectively reduce the risk factors for CVD, improve cardiovascular function, and provide direct endogenous cardiovascular protection, such as improving body metabolism and chronic inflammation, reducing blood pressure and fat, and improving cardiovascular flexibility (Leosco et al., 2013; Roh et al., 2016; Costa et al., 2018; Short et al., 2019; Taherkhani et al., 2020). These beneficial effects interact with each other to promote cardiovascular health. However, the mechanisms of the cardioprotective effect of exercise are still unclear. Exercise acts as a “polypill” for cardiovascular disease prevention and enables the body to have an endogenous “medkit” with unlimited refills of this marvelous polypill (Fiuza-Luces et al., 2013). Recent studies have (Akhmerov and Parimon, 2022) shown that exerkines secreted from multiple tissues in response to exercise orchestrate multiorgan cross-talk in exercise-induced cardiovascular benefits (Hawley et al., 2014). Clinically, exercise intervention has become a fundamental strategy in cardiac rehabilitation in recent years.

Exosomes, which are extracellular vesicles (EVs), are released to the outside of cells after the fusion of the multivesicular body (MVB) with the cell membrane and contain proteins, mRNA, miRNA, and DNA (Kalluri and LeBleu, 2020; Crescitelli et al., 2021). Recently, studies have provided insights into the exosomes of different cells, indicating that exosomes have the ability to target their parental cells (Qiao et al., 2020). It has been shown to play an essential role in mediating cardiovascular cell-to-cell crosstalk between organs via the transmission of various cargos, suggesting that suggests that exosomes may be important biomarkers in CVD (Li et al., 2017). In particular, the exosome cargos of proteins need a medium to be delivered from donor cells to recipient cells. However, the molecular mechanisms of exosome biogenesis, especially those exploited by stem cells in the cardiovascular system, have been less intensively investigated (Poe and Knowlton, 2018; Bellin et al., 2019). Interestingly, exosomes participate in the pathogenesis of CVD and are derived from the circRNA-0002113/miR-188-3-p/R-UNX1 signaling pathway, which mediates the alleviation of apoptosis and suppresses myocardial infarction (Tian et al., 2021). MiR-19a could suppress apoptosis of myocardial cells and was detected to be lower in myocardial tissues of acute myocardial infarction (AMI) compared to normal tissues, while human umbilical cord mesenchymal stem cell-derived exosomes (hucMSC-Exos) significantly increased the release of miR-19a and attenuated ischemic injury with decreased expression of inflammatory cytokines (Ma et al., 2020). In recent years, an increasing number of studies have focused on miRNAs carried by different exercise-induced exosomes, which can promote the proliferation of cardiomyocytes and protect the cardiovascular system (Del Campo et al., 2022; Liu et al., 2022). Studies have found that exercise affects the biological function of circulating exosomes, and different forms of exercise and exercise intensity can promote exosome release (Estébanez et al., 2021). D’Souza et al. and Yin et al. showed that the number of circulating exosomes and the expression of exosome-carried miR-1, miR-133a, miR-133b, miR-206, miR-208a, and miR-499 were elevated in response to exercise protocols and returned to baseline levels at 4–48 h into recovery (D'Souza et al., 2018; Yin et al., 2019). These findings indicate that exercise-induced release of exosomes into circulation may play a role in exercise-conferred systemic adaptations. In addition, exercise-derived exosomes have a wide variety of applications in the prevention and treatment of CVD, such as promoting angiogenesis and blood circulation through intercellular communication, inhibiting myocardial apoptosis, regulating the inflammatory response, repairing myocardial tissue, and improving cardiac function after myocardial infarction, thus playing a beneficial role in cardiovascular protection (Akhmerov and Parimon, 2022; Lee et al., 2022; Martin-Ventura et al., 2022). However, little is known about the role of exosomes in different exercise types (e.g., acute and chronicexercise, aerobic, among other types) and intensities (e.g., low, moderate, and high intensity) that induce cardioprotection.

When considering the importance of exercise intervention as a nonpharmacological means to prevent and treat CVD, a systematic evaluation and meta-analysis of preclinical studies on the therapeutic efficacy of exercise-induced exosomes in MI/R, I/R, and IS models have yet to be conducted. Therefore, this review aimed to systematically evaluate exosomes as a delivery tool of exercise-induced benefit factors of potential prevention and treatment for CVD and summarize these outcomes with an overview of their therapeutic implications.

## Materials and methods

### Literature search strategy

This study was performed according to the recommendations of the Cochrane Handbook for Systematic Reviews of Interventions and reported by following the Preferred Reporting Items for Systematic Reviews and Meta-Analyses (PRISMA) statement (Higgins et al., 2008; Moher et al., 2009). A systematic literature search was conducted in nine electronic databases, EBSCOhost, PubMed, Embase, Cochrane Central Register of Controlled Trials (CENTRAL), Ovid, Science Direct, Scopus, Wiley and Web of Science, from inception to 26 June 2022. At the same time, the references of the included literature were traced back to “snowball” to supplement the acquisition of relevant literature. For a comprehensive search strategy, relevant articles written in English were searched by using the following keywords: (exercise or physical exercise or training) and (extracellular vesicle or exosomes or exosomal) and (cardiovascular disease or acute myocardial infarction or heart failure or cardiomyopathy or blood-brain barrier or myocardial ischemia/reperfusion (MI/R) or angiogenesis or coronary vessels or cardiac modeling or cardioprotection or blood lipid metabolism or cardiomyocytes or endothelial cells or vascular smooth muscle cells or cardiovascular or atherosclerosis or coronary vessels or capillaries or cardiovascular). The bibliographies of identified articles were also manually checked, including relevant reviews and meta-analyses, to identify additional eligible studies.

### Study selection and eligibility criteria

Two reviewers (Z.J.L. and J.F.Z.) independently carried out the initial search, removed duplicate records, screened the titles and abstracts for relevance, and identified them as included, excluded, or uncertain. In case of uncertainty, the full-text article was reviewed to identify eligibility. Article selection was based on the following inclusion criteria in this systematic review and meta-analysis: 1) randomized controlled trials (RCTs) and nonrandomized controlled trials (non-RCTs); 2) the types of CVD included MI/R, myocardial infarction, heart failure, atherosclerosis of the coronary artery and blood brain barrier (BBB); 3) the effect of exercise on exosome release in tissue, plasma, serum, or other body fluids and the number or size and exosomal content (proteins or nucleic acids) were measured; 4) the relationship between exosome secretion level and the diagnosis and prognosis of CVD was reported in case-control studies or could be measured from the provided data; 5) studies that investigated the impact of exercise on exosome intervention on myocardial apoptosis, myocardial infarction volume, angiogenesis, microvessel density, and left ventricular ejection fraction; and 6) studies that presented outcome data of interest as the standardized mean difference (SMD) and 95% confidence interval (95% CI) in the treatment and control groups.

The exclusion criteria used for the article output were as follows: 1) no other intervention approach was implemented in addition to exercise and exosomes; 2) additional systematic disorders; 3) meeting abstracts, conference or congress communications, review articles, books or book chapters, project papers, editorials, letters, corrections, retractions, and comments; and 4) articles in a language other than English. Fifth, studies published more than 10 years.

### Data extraction

Data were extracted by two investigators (Z.J.L. and J.F.Z.) and confirmed by a third investigator (G.Q.X.) using a standardized electronic form. The following critical component data were collected from the included studies. First, first author name, year of publication, country, model characteristic (human, rat, mouse), sample size, injury model, physical activity information or exercise protocol. Second, the source of samples (plasma, serum, or cell super-natant), exosome number, size, and distribution, and level of exosome biomarkers and other exosome-contained proteins and RNAs (miRNAs, piRNAs, tRNAs, and cfDNA) were assessed. Third, isolation methodology (ExoQuick^TM^ and ultracentrifugation [UC]), characterization methodology (electron microscopy, flow cytometry [FCM], immunostaining, and nanoparticle tracking assay [NTA]), and phenotyping methodology (ELISA and Western blotting [WB]) were used. In addition, for studies with multiple time points, only the last endpoint was used for the statistical analysis.

### Quality assessment

Two reviewers (Z.J.L. and J.F.Z.) used the Cochrane Risk of Bias Assessment Tool to assess the risk and quality of bias in the selected RCTs (Higgins et al., 2008). Each study was reviewed and scored as having a high, low, or unclear risk of bias according to the following domains: random sequence generation, allocation concealment, blinding of participants and personnel, blinding of outcome assessment, incomplete outcome data, selective reporting, and other bias. The assessment was first performed independently by two authors, and when discrepancies were encountered, they were resolved by discussion until consensus was achieved with a third author.

### Statistical analysis

Meta-analysis was performed using STATA 16.0 (StataCorp, College Station, TX, United States) and RevMan 5.4 (The Cochrane Collaboration, Copenhagen, Denmark). Outcomes were carried out using random-effects models, which were used to calculate the effect size (DerSimonian and Kacker, 2007). The effect sizes of myocardial apoptosis, myocardial infarction volume, angiogenesis, microvessel density and left ventricular ejection fraction were estimated using standardized mean difference (SMD) and 95% CI, and the level of significance was set at *p* < 0.05. A forest plot was generated for each analysis to display the results of syntheses visually. The level of heterogeneity was set at *p* < 0.1, and the level of no heterogeneity was set at *p* > 0.1. Heterogeneity across studies was tested by using the I^2^ statistic (Higgins et al., 2003)^.^ For the I^2^ test, 25%, 50%, and 75% represented low, moderate, and high heterogeneity, respectively (Higgins et al., 2003).

Publication bias was assessed using funnel plots, Egger’s linear regression test and Begg’s rank correlation test; the results were considered publication bias at *p* < 0.05 (Egger et al., 1997). In addition, a sensitivity analysis was performed to test the validity of the meta-analysis, and studies with possible sources of uncertainty were excluded by screening out each specific study.

## Results

### Study selection

**FIGURE 1:**
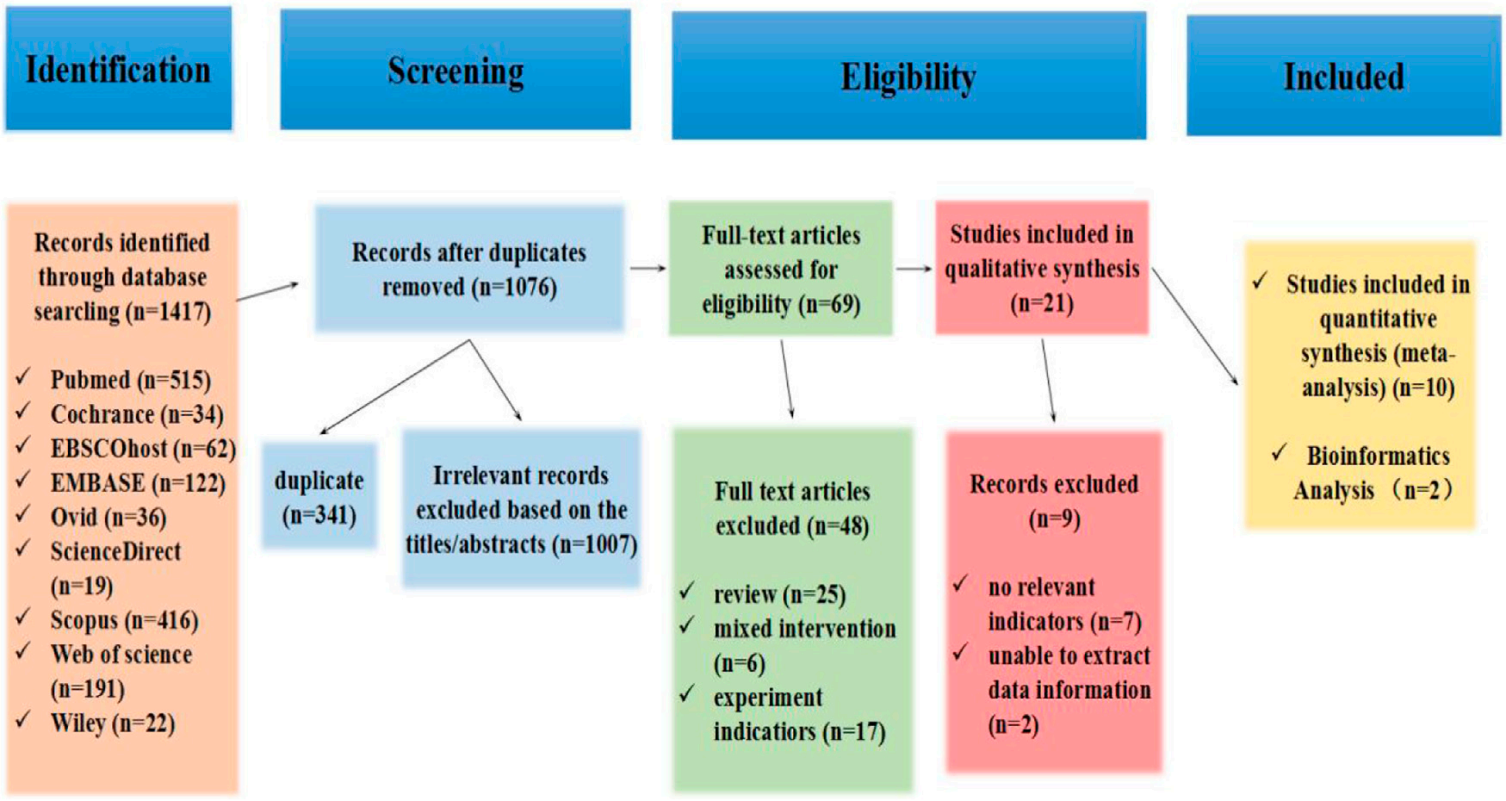
Flowchart of the study selection process in the systematic review according to PRISMA guidelines.

A total of 1417 articles were obtained in the database search. After removing duplicates (n = 341), the titles and abstracts of 1076 articles were filtered for eligibility (n = 1007). Moreover, the full texts of 69 studies remained for a detailed evaluation. Among them, 6 articles did not match the intervention approach, 25 reviews, and 17 did not match the experiment indicators. Subsequently, qualitative synthesis of 21 articles remained for a detailed evaluation; records excluded 7 articles that did not have relevant indicators, and 2 articles could not extract data information. Finally, after reviewing the complete text, 12 studies were considered eligible for this systematic review and meta-analysis (Chaturvedi et al., 2015; Wilhelm et al., 2016; Bei et al., 2017; Ma et al., 2018; Oliveira et al., 2018; Hou et al., 2019; Nie et al., 2019; Wang et al., 2020; Huang et al., 2021; Kränkel et al., 2020; Lou et al., 2021; Zhao et al., 2022). The study flow diagram, including the reasons for the exclusion of studies, is shown in Figure 1.

### Study characteristics

The characteristics of 10 RCTs, including research articles, are summarized in Table 1. A total of 104 sample sizes were included in the analysis, and only males were enrolled in all articles. Moreover, only mice were enrolled as subjects in six articles (Chaturvedi et al., 2015; Ma et al., 2018; Nie et al., 2019; Wang et al., 2020; Lou et al., 2021; Zhao et al., 2022), only humans were enrolled as participants in two articles (Wilhelm et al., 2016; Kränkel et al., 2020), and mice and humans were enrolled as subjects in two articles (Bei et al., 2017; Hou et al., 2019). The studies were conducted in some injury models, such as MI/R injury (Hou et al., 2019; Zhao et al., 2022), middle cerebral artery occlusion (MCAO) (Wang et al., 2020), MCAO-induced ischemic stroke (IS) (Wang et al., 2020), hypoxia/reoxygenation (H/R) injury (Hou et al., 2019; Wang et al., 2020), chronic coronary syndrome (CCS) (Kränkel et al., 2020), ischemia-reperfusion (I/R) injury (Bei et al., 2017), high glucose/hypoxia (HG/H) (Ma et al., 2018), and type 2 diabetes (T2DM) (Chaturvedi et al., 2015). These studies used the primary type of aerobic exercise as the protocol, especially swimming and running. Additionally, all the studies were published within the last 10 years, between 2015 and 2022. Moreover, six articles were conducted from China (Bei et al., 2017; Ma et al., 2018; Hou et al., 2019; Wang et al., 2020; Lou et al., 2021; Zhao et al., 2022), two articles from America (Chaturvedi et al., 2015; Nie et al., 2019), one article from Germany (Kränkel et al., 2020), and one article from Britain (Wilhelm et al., 2016).

**TABLE 1:** Characteristics of the articles included in the meta-analysis.

Research article	Subject characteristic	Gender (F/M)	Injury model	Exercise protocol	Results
Zhao et al. (2022, China)	Mouse (C57BL/6, 8–10 wk old, n = 12)	M	MI/R	4-wks swimming (90 min*2/day)	Left ventricular ejection fraction
					Myocardial infarction volume
					Myocardial apoptosis
Lou et al. (2021, China)	Mouse (C57BL/6, n = 12)	M	/	9-days incremental cycling treadmill training (18 m/min, 1 h/day)	Microvessel density
					Angiogenesis
Wang et al. (2020, China)	Mouse (C57BL/6, 10 wk old, n = 11)	M	MCAO, IS, H/R	4-wks treadmill exercise (10 m/min, 1 h/day, 5days/wk)	Myocardial infarction volume
					Myocardial apoptosis
					Microvessel density
Kränkel et al. (2020, Germany)	Human (n = 12)	M	CCS	4-wks HIIT	Myocardial apoptosis
Nie et al. (2019, United States)	Mouse (C57BL/6J, 8–10 wk old)	M	C2C12	/	Myocardial apoptosis
					Angiogenesis
Hou et al. (2019, China)	1. Rats (Sprague‒Dawley, 6 wk old, n = 8)	M	MI/R, H/R	1. 4-wks swimming (90 min*2/day, 7-days/wk)	Myocardial apoptosis
	2. Human (n = 32)			2. 1-year rowing	Left ventricular ejection fraction
					Myocardial infarction volume
Bei et al. (2017, China)	1. Human (n = 16)	M	I/R	1. Bruce stress test (running)	Myocardial infarction volume
	2. Mouse (C57BL/6, 8 wk old, n = 4)			2. 3-wks swimming (90 min*2/day)	Myocardial apoptosis
MA et al. (2018, China)	Mouse (C57BL/6, 8–10 wk old, n = 12–18)	M	HG/H	4-wks treadmill running (5–10 m/min, 60 min/day, 5-days/wk)	Myocardial apoptosis
					Angiogenesis
Wilhelm et al. (2016, United Kingdom)	Human (n = 9)	M	/	incremental running	Angiogenesis
Chaturvedi et al. (2015, United States)	Mouse (db/db)	M	T2DM	8-wks treadmill running (7–10 m/min, 300 m/day, 5-days/wk)	Fibrosis
					Myocyte uncoupling

wk, week; F/M, Female/male; MI/R, Myocardial ischemia/reperfusion injury; MCAO, middle cerebral artery occlusion; IS, MCAO-induced ischemic stroke; H/R, Hypoxia/reoxygenation injury; CCS, chronic coronary syndrome; I/R, Ischemia–reperfusion injury; HG/H, High glucose/hypoxia; T2DM, Type 2 diabetes; HIIT, High-intensity interval exercise training.

As shown in Table 2, these articles were conducted with five outcome indicators: myocardial apoptosis (Bei et al., 2017; Ma et al., 2018; Hou et al., 2019; Nie et al., 2019; Wang et al., 2020; Kränkel et al., 2020; Zhao et al., 2022), myocardial infarction volume (Bei et al., 2017; Hou et al., 2019; Wang et al., 2020; Zhao et al., 2022), angiogenesis (Wilhelm et al., 2016; Ma et al., 2018; Nie et al., 2019; Lou et al., 2021), microvessel density (Wang et al., 2020; Lou et al., 2021), and left ventricular ejection fraction (Hou et al., 2019; Zhao et al., 2022). Moreover, one article did not include these outcome indicators, but exercise-induced exosomes inhibit fibrosis and myocyte uncoupling.

**TABLE 2:** Outcomes of studies included in the meta-analysis.

Research article	Myocardial apoptosis	Myocardial infarction volume	Angiogenesis	Microvessel density	Left ventricular ejection fraction
Zhao et al. (2022, China)	√	√			√
Lou et al. (2021, China)			√	√	
Wang et al. (2020, China)	√	√		√	
Kränkel et al. (2020, Germany)	√				
Nie et al. (2019, United States)	√		√		
Hou et al. (2019, China)	√	√			√
Bei et al. (2017,China)	√	√			
MA et al. (2018, China)	√		√		
Wilhelm et al. (2016, United Kingdom)			√		
Chaturvedi et al. (2015, United States)	Fibrosis and myocyte uncoupling				

### Results from quality assessments

**FIGURE 2:**
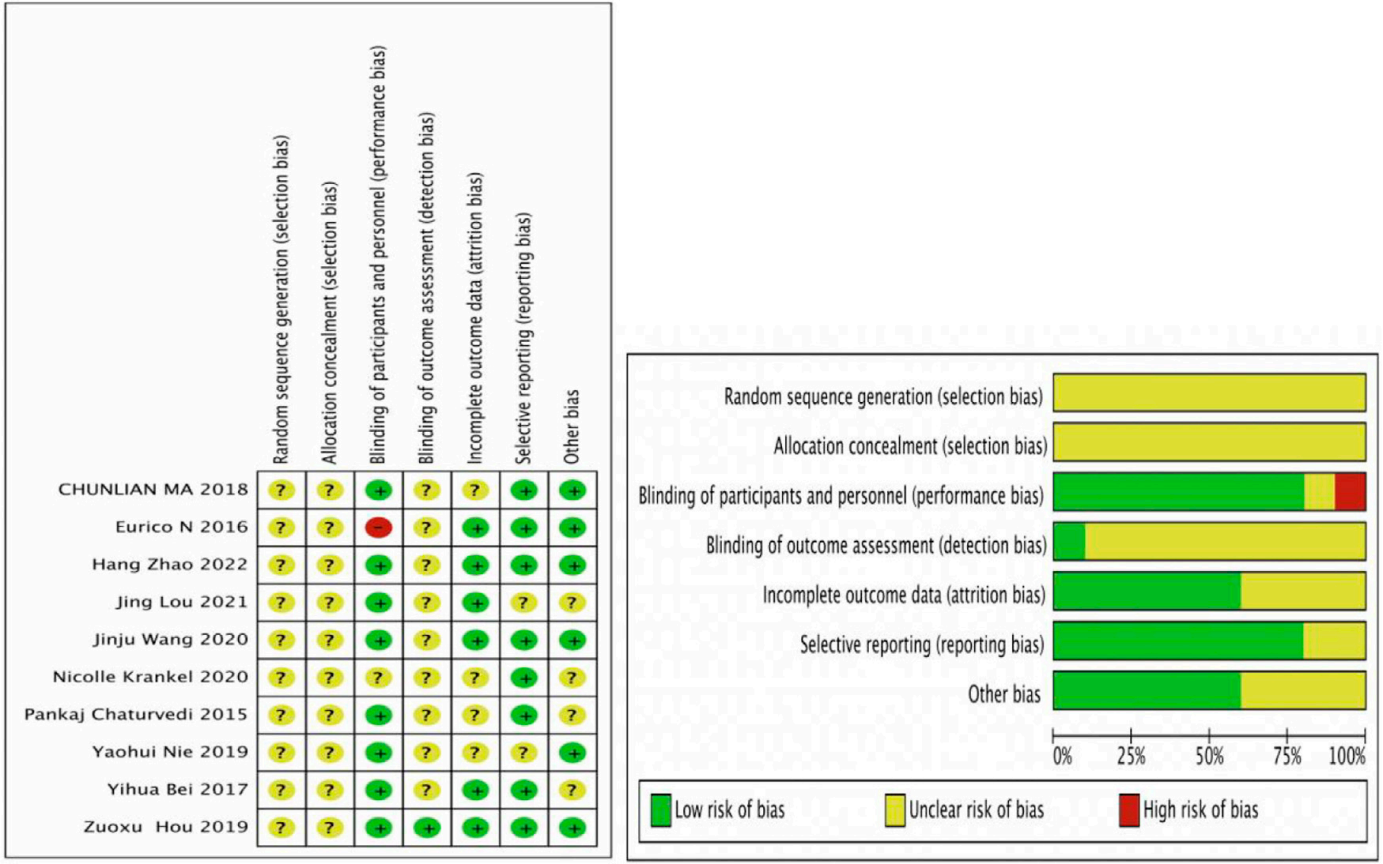
Risk of bias results. Review authors’ judgments about each risk of bias item for each included study. +, low risk of bias; -, high risk of bias; ?, unclear risk of bias.

The Cochrane Risk of Bias tool to assess the randomized controlled trials (RCTs), which were found to have a high quality among the 10 RCTs, is shown in Figure 2. Ten studies could have been described more clearly in random sequence generation and allocation concealment. For blinding, seven studies were single-blind experiments that blinded the operators (Chaturvedi et al., 2015; Bei et al., 2017; Ma et al., 2018; Nie et al., 2019; Wang et al., 2020; Lou et al., 2021; Zhao et al., 2022), one study was a double-blind experiment that blinded the operator and outcome indicator (Hou et al., 2019), and one study clearly described that the participants knew the content of the experiment (Wilhelm et al., 2016). In contrast, another study could have been described more clearly (Kränkel et al., 2020). For incomplete outcome data, six studies had complete outcome data (Bei et al., 2017; Hou et al., 2019; Nie et al., 2019; Wang et al., 2020; Lou et al., 2021; Zhao et al., 2022), and four studies were described unclearly (Chaturvedi et al., 2015; Ma et al., 2018; Nie et al., 2019; Kränkel et al., 2020). Eight studies were selective reporting (Chaturvedi et al., 2015; Wilhelm et al., 2016; Bei et al., 2017; Ma et al., 2018; Hou et al., 2019; Wang et al., 2020; Kränkel et al., 2020; Zhao et al., 2022), and two studies were described unclearly (Nie et al., 2019; Lou et al., 2021). For other biases, six studies had no other bias (Wilhelm et al., 2016; Ma et al., 2018; Hou et al., 2019; Nie et al., 2019; Wang et al., 2020; Zhao et al., 2022), and other studies were described unclearly (Chaturvedi et al., 2015; Bei et al., 2017; Kränkel et al., 2020; Lou et al., 2021).

### Primary outcome measures

**FIGURE 3:**
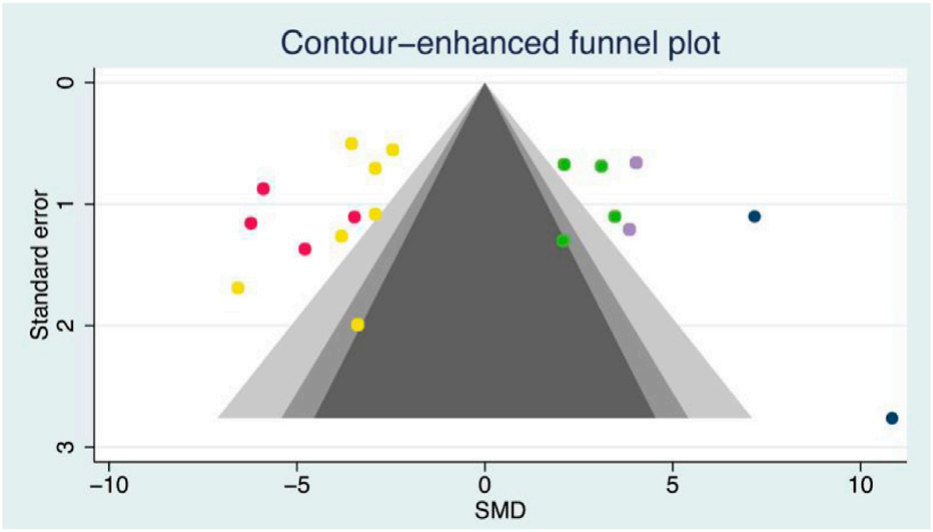
Contour-enhanced funnel plot of outcome indicators. Yellow dot, A total of seven articles provided relevant data on the myocardial apoptosis (n = 7); red dot, A total of four articles provided relevant data on the myocardial infarction volume (n = 4); green dot, A total of four articles provided relevant data on the angiogenesis (n = 4); purple dot, A total of two articles provided relevant data on the microvessel density (n = 2); blue dot, A total of two articles provided relevant data on the left ventricular ejection fraction (n = 2). 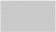, 1% < *p* < 5%; 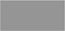, 5% < *p* < 10%; 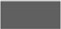, *p* > 10%; 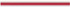, Estimated θ_Ⅳ_.

**FIGURE 4:**
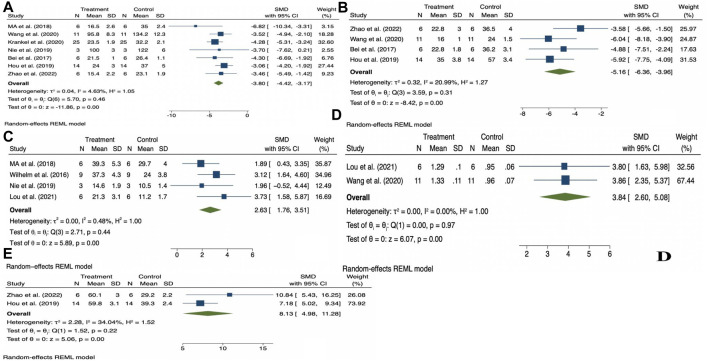
Forest plot of outcome indicators **(A)**, Myocardial apoptosis; **(B)**, Myocardial infarction volume; **(C)**, Angiogenesis; **(D)**, Microvessel density; **(E)**, Left ventricular ejection fraction.

The random effects model was used for the overall analysis to assess differences in the pooled effect. As shown in Figure 3, there was no significant asymmetry, and Egger’s and Begg’s tests showed that *p >* 0.05, demonstrating that no publication bias was produced for all outcomes. As shown in Table 3; Figure 4, exercise-induced exosome release significantly reduced myocardial apoptosis (N = 7, SMD = −3.80, 95% CI = [−4.42, −3.17], *p* = 0.46, I^2^ = 4.63%), and the I^2^ value indicated low statistical heterogeneity (Bei et al., 2017; Ma et al., 2018; Hou et al., 2019; Nie et al., 2019; Wang et al., 2020; Kränkel et al., 2020; Zhao et al., 2022). Moreover, it significantly reduced myocardial infarction volume (N = 4, SMD = −5.16, 95% CI = [−6.36, −3.96], *p* = 0.31, I^2^ = 20.99%), and the I^2^ value indicated low statistical heterogeneity (Bei et al., 2017; Hou et al., 2019; Wang et al., 2020; Zhao et al., 2022). However, it significantly increased angiogenesis (N = 4, SMD = 2.63, 95% CI = [1.76, 3.51], *p* = 0.44, I^2^ = 0.48%), and the I^2^ value indicated low statistical heterogeneity (Wilhelm et al., 2016; Ma et al., 2018; Nie et al., 2019; Lou et al., 2021). It can significantly increase microvessel density (N = 2, SMD = 3.84, 95% CI = [2.60, 5.08], *p* = 0.97, I^2^ = 0.00%), and the I^2^ value indicates low statistical heterogeneity (Wang et al., 2020; Lou et al., 2021). In addition, it significantly increased the left ventricular ejection fraction (N = 2, SMD = 8.13, 95% CI = [4.98, 11.28], *p* = 0.22, I^2^ = 34.04%), and the I^2^ value indicated low statistical heterogeneity (Hou et al., 2019; Zhao et al., 2022). In addition, any individual effect size had no significant effect on all the parameters’ overall effect size.

**TABLE 3:** Effects of exercise on exosome intervention on primary outcome measures.

Outocmes	Research article	Treatment	Control	SMD	95%CI	I^2^ (%)	*p*
Myocardial apoptosis	Zhao et al. (2022)	15.40 ± 2.20	23.10 ± 1.90	−3.46	−5.49, 1.42		
	Wang et al. (2020)	95.80 ± 8.30	134.20 ± 12.30	−3.52	−4.94, −2.10		
	Kränkel et al. (2020)	23.50 ± 1.90	32.20 ± 2.10	−4.28	−5.31, −3.24		
	Nie et al. (2019)	100.00 ± 3.00	122.00 ± 6.00	−3.70	−7.62, 0.21		
	Hou et al. (2019)	24.00 ± 3.00	37.00 ± 5.00	−3.06	−4.20, −1.92		
	MA et al. (2018)	16.50 ± 2.60	35.00 ± 2.40	−6.82	−10.34, −3.31		
	Bei et al. (2017)	21.50 ± 1.00	26.40 ± 1.10	−4.30	−6.69, −1.92		
	overall			−3.80	−4.42, −3.17	4.63	0.46
Myocardial infarction volume	Zhao et al. (2022)	22.80 ± 3.00	36.50 ± 4.00	−3.58	−5.66, −1.50		
	Wang et al. (2020)	16.00 ± 1.00	24.00 ± 1.50	−6.04	−8.18, −3.90		
	Hou et al. (2019)	35.00 ± 3.80	57.00 ± 3.40	−5.92	−7.75, −4.09		
	Bei et al. (2017)	22.80 ± 1.80	36.20 ± 3.10	−4.88	−7.51, −2.24		
	overall			−5.16	−6.36, −3.96	20.99	0.31
Angiogen -esis	Lou et al. (2021)	21.30 ± 3.10	11.20 ± 1.70	3.73	1.58, 5.87		
	Nie et al. (2019)	14.60 ± 1.90	10.50 ± 1.40	1.96	−0.52, 4.44		
	MA et al. (2018)	39.30 ± 5.30	29.70 ± 4.00	1.89	0.43, 3.35		
	Wilhelm et al. (2016)	37.30 ± 4.30	24.00 ± 3.80	3.12	1.64, 4.60		
	overall			2.63	1.76, 3.51	0.48	0.44
Microvessel density	Lou et al. (2021)	1.29 ± 0.10	0.95 ± 0.06	3.80	1.63, 5.98		
	Wang et al. (2020)	1.33 ± 0.11	0.96 ± 0.07	3.86	2.35, 5.37		
	overall			3.84	2.60, 5.08	0.00	0.97
Left ventricular ejection fraction	Zhao et al. (2022)	60.10 ± 3.00	29.20 ± 2.20	10.84	5.43, 16.25		
	Hou et al. (2019)	59.80 ± 3.10	39.30 ± 2.40	8.13	5.02, 9.34		
	overall			8.13	4.98, 11.28	34.04	0.22

### Basic characteristics of exercise-derived exosomes and proposed mechanism of exosome-carried cargos

**FIGURE 5:**
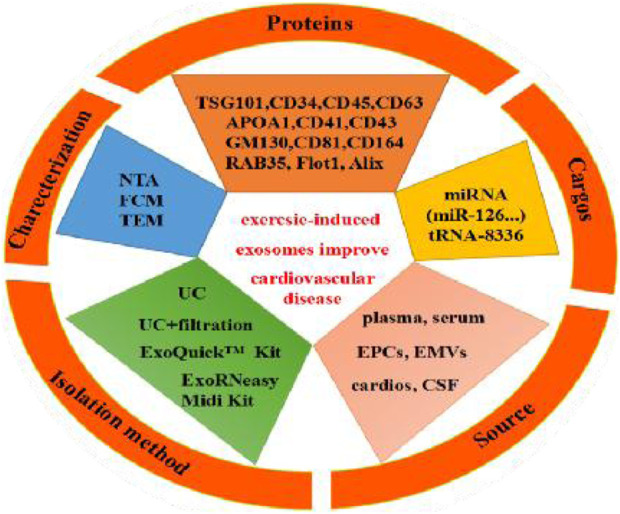
Exercise-induced release of exosome source, isolation method, characterization, exosome markers (proteins) and exosome-carried cargos.

The characteristics of exosomes and the impact of exercise-derived exosomes in human, animal, and cell culture subjects are summarized in Table 4 and shown Figure 5. Specifically, the source, size, distribution, marker cargos, and mechanism of exosomes following different exercise protocols were evaluated in ten research articles. Six studies examined exosome sources of plasma (Bei et al., 2017; Hou et al., 2019; Wang et al., 2020; Kränkel et al., 2020; Lou et al., 2021; Zhao et al., 2022), two studies examined exosome sources of serum (Bei et al., 2017; Nie et al., 2019), and other studies examined sources of endothelial progenitor cells (EPCs) (Ma et al., 2018), endothelial-derived microvesicles (EMVs) (Wilhelm et al., 2016), and exosomes from cardiomyocytes (cardiosomes) (Chaturvedi et al., 2015). Moreover, the results of this study showed concordance with preceding studies that ultracentrifugation (UC) was the most widely adopted method to isolate exosomes. Six studies used UC for isolation (Chaturvedi et al., 2015; Bei et al., 2017; Nie et al., 2019; Wang et al., 2020; Lou et al., 2021; Zhao et al., 2022), and filtration was combined with UC to remove high-abundance proteins in two studies (Nie et al., 2019; Kränkel et al., 2020). In addition, ExoQuick^TM^ commercial reagent was chosen in two studies (Bei et al., 2017; Hou et al., 2019), and magnetically activated cell sorting was chosen in one study (Ma et al., 2018). However, regardless of the isolation methods utilized, the consensus reached among researchers was that a combination of at least one visualization method and one physical technique should be utilized to quantify and characterize exosome markers. Additionally, all studies identified that the diameter range of exosomes was 30–200 nm in eight studies using the nanoparticle tracking assay (NTA) technique to characterize the size of exosomes (Bei et al., 2017; Ma et al., 2018; Hou et al., 2019; Nie et al., 2019; Wang et al., 2020; Kränkel et al., 2020; Lou et al., 2021; Zhao et al., 2022), and other studies used the flow cytometry (FCM) technique (Chaturvedi et al., 2015; Bei et al., 2017).

**TABLE 4:** Characteristics of studies evaluating exosome/EV size and markers and exosome-carried cargos in response to exercise protocols in the meta-analysis.

Research article	Subject	Exercise protocol		Exosome/EV source	Isolation method	Characterization method	Size	Phenotyping method	Exosome/EV markers	Exosome-carried cargos	Proposed mechanism	Beneficial effects
Zhao et al. (2022)	Mouse	4-wks swimming		Plasma	UC	NTA	80–120 nm	WB	/	miR-125b-5p	MAPK (Map3k5、Map2k7、 Map2k4) (−) Caspase-3 (−)	Mediating cardioprotection against MI/R injury
										miR-128-3p		
										miR-30d-5p		
Lou et al. (2021)	Mouse	9-days running		Plasma	UC	NTA	100 nm	WB	TSG101	miR-122-5p	(↓)AGPAT1	Promoting angiogenesis and accelerate wound healing
									CD81		(↑)CD31	
									APOA1		(↑)VEGF	
									GM130			
Wang et al. (2020)	Mouse	4-wks running		Plasma	UC	NTA	100–150 nm	WB	CD34	miR-126	(↓)PI3k (−)	Protecting effects on the brain against MCAO-induced ischemic injury in both acute and chronic stages
											(↑) BDNF	
											(↑) TrkB	
											(↑) Akt	
Kränkel et al. (2020)	Human	4-wks HIIT		Plasma	UC + filtration	NTA	150–200 nm	WB	CD41	miR-15a	VCAM-1	Mediating endothelial repair and therapy to improve endothelial function
									CD45	miR-20a		
									CD164	miR-21		
										miR-197		
Nie et al. (2019)	Mouse	/		serum	UC + filtration	NTA	30–200 nm	WB	Alix、CD63 TSG101	miR-130a	(↑)NF-kB	Providing an effective therapy for promoting skeletal muscle angiogenesis in diseases
Hou et al. (2019)	1.Rat	1. 4-weeks swimming		Plasma	UC + ExoQuick	NTA	100–150 nm	WB	CD81	miR-342-5p	1. Caspase-9 (−) Jnk2 (−)	Protecting the heart against MI/R injury and cardioprotection.
	2.Human	2. 1-year rowing							TSG101		2. Ppmlf (−) p-Akt (+)	
Bei et al. (2017)	1.Human	1. running		Plasma serum	Whole plasma	Nano-FCM	100 nm	WB	CD63	ALIX	1. ERK1/2 (+)	Protecting against myocardial injury
	2.Mouse	2. 3-wks swimming			ExoQuick™	NTA				RAB35	2. HSP27(+)	
					UC							
MA et al. (2018)	Mouse	4-wks running		EPCs	MACS	NTA	100–120 nm	WB	CD63	miR-126	(↑)VEGF, (↓)SPRED1	Enhancing the effects on protecting EC against injury.
									TSG101			
Wilhelm et al. (2016)	Human	running		EMVs	UC	Nano	100 nm	WB	CD43	/	(↑)VEGF	Mediating vascular healing and adaptation.
Chaturvedi et al. (2015)	Mouse	8-wks running		cardiosomes	UC	Nano-FCM	60–90 nm	WB	Flot1	miR-29b	(↓)MMP-9	Decreasing fibrosis and myocyte uncoupling.
									CD81	miR-455		

Wk, week; EPCs, endothelial progenitor cells; EMVs, endothelial-derived microvesicles; UC, ultracentrifugation; NTA, nanoparticle tracking assay; FCM, flow cytometry; MACS, magnetic activated cell sorting; MAPK, Mitogen-associated protein kinase; AGPAT1,1-acyl-sn-glycerol-3-phosphate acyltransferase; EVs, Extracellular vesicles; HIIT, High-intensity interval exercise training; cardiosomes, exosomes from cardiomyocytes; WB, Western blotting; NF-kB, Nuclear factor-kB; VEGF, vascular endothelial growth factor; PI3k, Phosphatidylinositol 3-kinase; BDNF, Brain-derived neurotrophic factor; TrkB, Tyrosine kinase receptor B; akt, Protein kinase B; VCAM-1, Vascular cell adhesion molecule-1; Jnk2, c-Jun N-terminal kinase; ERK1/2, extracellular regulated protein kinases; HSP27, Heat shock proteins 27; SPRED1, Sprouty related EVH1 domain containing 1; MMP-9, Matrix metalloprotein-9.

**FIGURE 6:**
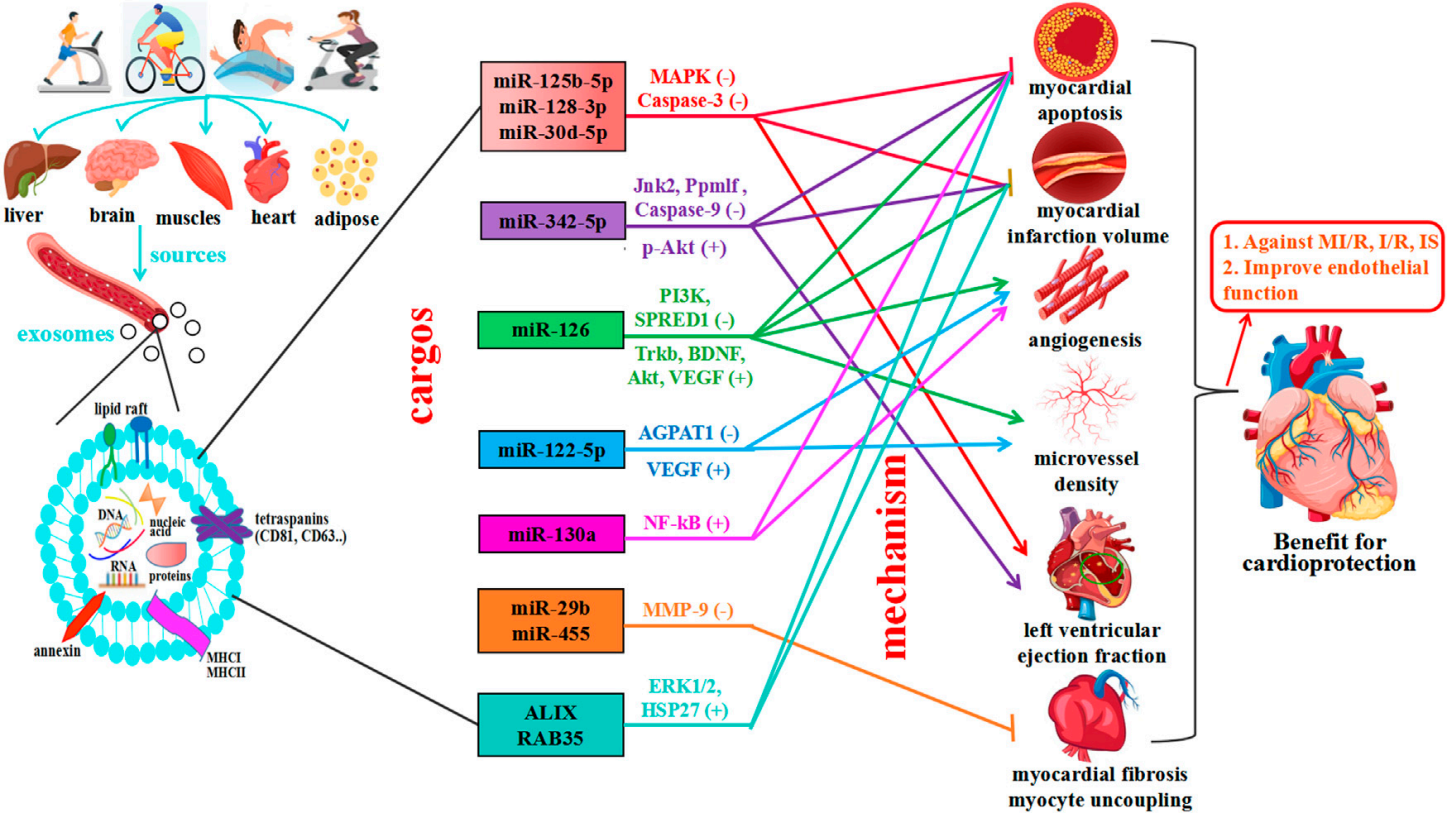
Exercise induces multiple tissues to release exosomes, which mediate cardiovascular protection. The main effects of exercise on exosome source of release, which can derive from different organs (liver, brain, muscle, heart, and adipose tissue), cargo, and exercise-released exosome-carried miRNAs predicted actions. Exercise promotes cardiovascular health by mediating cell-to-cell communication and cross-talk between organs and tissues—exosomes are significant vehicles for miRNAs and proteins.

Finally, 9 articles were included to use Western blotting (WB) to investigate exosomal markers in response to exercise and assess the effects of exercise on 13 exosome-carried proteins (TSG101, APOA1, GM130, CD34, CD41, CD43, CD45, CD81, CD63, CD164, Flot1, Alix, RAB35). Cell surface protein characterization is a vital step in identifying exosomes. Moreover, exercise regulates the expression of 13 exosomal miRNAs (miR-125b-5p, miR-128-3p, miR-30d-5p, miR-122-5p, miR-126, miR-15a, miR-20a, miR-21, miR-197, miR-130a, miR-342-5p, miR-29b, and miR-455); these exosomal miRNAs were significantly upregulated, and the majority of studies reported no overlapping miRNAs. Interestingly, two independent studies reported the same exosome cargo: miR-126 (Ma et al., 2018; Wang et al., 2020). Notably, these independent studies proposed variable mechanisms by which miR-126 influences cardioprotection. One study assessed the mechanism of action: moderate aerobic exercise induces endothelial progenitor cell-derived exosomal miR-126 to modulate the PI3K/Akt signaling pathway, increasing the expression of proteins brain-derived neurotrophic factor (BDNF) and tyrosine kinase receptor B (TrkB) to stimulate angiogenesis and enhance neurogenesis in the chronic phase, thereby reducing I/R-induced ischemic injury. Another study assessed the mechanism by which miR-126 promotes vascular repair and angiogenesis to protect ECs against injury through sprout-related EVH1 domain containing 1 (SPRED1) downregulation and vascular endothelial growth factor (VEGF) upregulation. These factors can enhance the effects against ischemic injury. In addition, all other studies posited a regulatory mechanism of action that improved CVD, as shown in Figure 6.

### Basic characteristics of exercise-derived exosomes and exosome-carried cargo bioinformatic analysis

Only two studies reported exosome-carried cargos for bioinformatics analysis in response to exercise protocols, and the characteristics of these studies are summarized in Table 5. Exosomes marked with CD63 were derived from cerebrospinal fluid (CSF) and serum. Interestingly, studies have observed the expression of various miRNAs in exosome-carried cargos following exercise. For example, Huang et al. showed that miR-370-3p, miR-343, miR-92b-5p, miR-138-5p, and miR-34c-5p were upregulated after exercise, and the levels of miR-665, miR-3573-5p, miR-1188-5p, miR-31a-5p, and miR-23b-5p were downregulated after exercise (Huang et al., 2021). These miRNA target genes are mainly involved in the most enriched categories, including cellular response to retinoic acid, vagus nerve morphogenesis, cellular response to hypoxia, dendritic cell chemotaxis, cell differentiation, and regulation of neuron death, which is related to axon guidance, nuclear factor-kB (NF-kB), thiamine metabolism, and mitogen-associated protein kinase (MAPK). In addition, this study independently proposed the mechanisms by which miR-138-5p influences cardioprotection. It not only suppressed inflammatory response states in astrocytes following ischemic stroke by targeting lipocalin 2 (LCN2) but also inhibited the target of caspase-9 and apoptosis-related cysteine peptidase (Casp9) against ischemic stroke (IS) injury.

**TABLE 5:** Methodology of exosome/EV isolation and characterization and exosome/EV cargo bioinformation in response to exercise protocols in studies.

Research article	Models	Exercise protocol	Source and solation and characterization	Exosome/EV-carried cargos	Bioinformation	Proposed mechanism
Huang et al. (2021, China)	MCAO and IS injury rats (SD, n = 6)	4-wks incremental running (2,3.25,6.59 m/min, 6days/wk)	CSF, ExoRNeasy Midi Kit, WB (CD63, CD81), NTA (30–100 nm)	↑: miR-370-3p, miR-343, miR-92b-5p, miR-138-5p, miR-34c-5p	GO: cellular response to retinoic acid, vagus nerve morphogenesis, cellular response to hypoxia, dendritic cell chemotaxis, cell differentiation, and regulation of neuron death	1. miR-138-5p → LCN2(−)→ inflammation→ against I/S injury.
				↓: miR-665, miR-3573-5p, miR-1188-5p, miR-31a-5p, miR-23b-5p	KEGG: axon guidance, NF-kB thiamine metabolism, MAPK	2. miR-138-5p → Caspase 9 → apoptosis-related cysteine peptidase → against I/S injury.
Oliveira et al. (2018, Brazil)	Wistar rats (n = 4–5)	Low, moderate and high intensity acute exercise (14–16 m/min, 20–22 m/min, 24–26 m/min)	Serum, UC + Exoquick^TM^ Kit, WB (CD63), TEM (40–200 nm)	tRNA-8336	MAPK, PDGFRA, MKNK2, NFATE3, TGFBR1, NLK	1. 12miRNAs → MAPK(−) → cardioprotection.
				↑: miR-330-5p, miR-10b-5p, miR-142-3p, miR-410-3p		2. miR-103-3p → CCL3(−)→ decrease the risk of cardiac diseases.
				↓: miR-128-3p, miR-103-3p, miR-148a-3p, miR-191a-5p, miR-93-5p, miR-25-3p, miR-142-5p, miR-3068-3p		3. miR-10b-5p → mib 1 and Noth(−)→ angiogenesis.

EV, extracellular vesicle; SD, Sprague‒Dawley; MCAO, middle cerebral artery occlusion; IS, MCAO-induced ischemic stroke; CSF, cerebrospinal fluid; NF-kB, Nuclear factor-kB; MAPK, Mitogen-associated protein kinase; Casp9, Caspase 9, Apoptosis-related cysteine peptidase; PDGFRA, platelet derived growth factor receptor beta; MKNK2, MAP, Kinase-interacting serine/threonine kinase 2; NFATE3, Nuclear factor of activated T cells 3; TGFBR1, Transforming growth factor beta receptor 1; NLK, nemo like kinase.

Another study showed that the concentration of tRNA-8336 in circulating EVs was elevated in response to incremental running (Oliveira et al., 2018), the levels of miR-330-5p, miR-10b-5p, miR-142-3p, and miR-410-3p were upregulated after exercise, and the levels of miR-128-3p, miR-103-3p, miR-148a-3p, miR-191a-5p, miR-93-5p, miR-25-3p, miR-142-5p, and miR-3068-3p were downregulated after exercise. These miRNA target genes were mainly related to MAPK, PDGFRA, MKNK2, NFATE3, TGFBR1, and NLK. In addition, these miRNAs inhibited the target of MAPK and the chemokine CCL3 to decrease the risk of CVD, and the mechanisms by which miR-10b-5p targets mib1 and Notch signaling to promote angiogenesis were proposed.

## Discussion

The primary purpose of the systematic review and meta-analysis of preclinical studies is to evaluate the role of exercise-induced exosomes in the prevention and treatment of CVD, while the secondary purpose is to provide guidance for the selection of different types of exercise-induced exosomes for further preclinical research. We extracted and compiled data from the research articles and provided a reference for the research design of preclinical cardioprotection and prevention of ischemic injury using exercise-induced exosome therapy. The main findings of this study show that myocardial apoptosis and myocardial infarction volume are significantly reduced, and angiogenesis microvessel density and left ventricular ejection fraction were significantly increased following the release of exercise-induced exosomes. These results provide a theoretical basis for the prevention and treatment of CVD. Moreover, funnel plot analysis showed that the studies were evenly distributed for the regulation of exercise exosomes in cardiovascular protection, suggesting no inherent bias in this study. Our meta-analysis and results further confirmed that exercise-derived exosomes had a higher target efficiency in cardiovascular tissue repair than other types of cells and supported the use of exercise-derived exosomes in clinical settings.

In recent years, studies have shown that exercise significantly impacts the biological function of body fluids and blood circulation exosomes (Pluchino and Smith, 2019). Moreover, different modes of exercise can also stimulate the release of exosomes and affect the quantity of these miRNAs and proteins, but exosome cargos vary among miRNAs, lncRNAs, and proteins (Emanueli, C., et al., 2015; Chang and Wang, 2019; Vicencio, et al., 2015; Estébanez et al., 2021). The systematic review and meta-analysis showed that moderate exercise promotes the release of exosomes and regulates the expression of miRNAs and proteins, which play critical roles in inhibiting myocardial apoptosis, decreasing myocardial infarction volume, promoting angiogenesis, increasing microvessel density, inhibiting myocardial fibrosis and protecting endothelial cells, providing a theoretical basis for the prevention and treatment of CVD, as shown Figure 5. Further study found that exercise-derived exosomes can enhance endogenous protection of cardiomyocytes against I/R injury and represent a promising alternative therapy against ischemic diseases (Beltrán-Camacho et al., 2021). First, exosomal miRNAs (BAT miRNAs) miR-125b-5p, miR-128-3p, and miR-30d-5p derived from brown adipose tissue target the proapoptotic MAPK (Map3k5, Map2k7, and Map2k4) and Caspase-3 pathways to suppress MI/R (Zhao et al., 2022). Exosomal miR-125a-5p targets endothelin converting enzyme 1 (ECE1) and activates the downstream AKT/eNOS signaling pathway to promote revascularization (Qiu et al., 2022). Second, long-term exercise-derived exosomal miR-342-5p not only targets the Caspase-9 and JNK2 pathways but also enhances survival signaling (p-Akt) by targeting the phosphatase gene Ppm1f to protect the heart from MI/R injury (Hou et al., 2019). From the same gene family, miR-342-3p has also been proven to participate in the regulatory processes related to acute myocardial infarction (AMI) by increasing the expression of NFAT activating molecule 1 (NFAM1) to combat myocardial injury (Zhu et al., 2022). Third, the expression of exercise-derived EV proteins ALIX and RAB35 activates ERK1/2 and HSP27 signals to protect against cardiac I/R injury (Bei et al., 2017). Recent research has highlighted the significant potential of endothelial cell (EC)-derived exosomal Profilin 2 (PFN2) as a valuable target in the repair of MI injury via angiogenesis, which promotes EC proliferation, migration, and tube formation through the PI3K-PFN2-ERK axis (Li, Z., et al., 2022). PFN2-enriched exosomes derived from ECs are intracellular proteins that promote angiogenesis *in vivo* and *in vitro*, repair EC injury under inflammatory stimuli, and significantly attenuate MI injury. However, in contrast to exosomal PFN2, PFN2 protein could not directly increase the viability and migration of ECs, implying that exosomes are critical mediators of PFN2-mediated angiogenic ability.

In addition, exercise-derived exosomes can promote revascularization, thus protecting the cardiovascular system. As a potential marker of transient ischemic attack (TIA), miR-122-5p targets OCLN to regulate the apoptosis and permeability of brain microvascular endothelial cells (BMECs) (Li et al., 2021; Lou et al., 2021). Lou et al. showed that exercise-derived EV miR-122-5p upregulated endothelial cell fatty acid utilization by targeting 1-acyl-sn-glycerol-3-phosphate acyltransferase (AGPAT1) and improving the impression of VEGF to promote angiogenesis (Zhao et al., 2022). Nie et al. also found that skeletal muscle-derived exosome (SkM-Exo) miR-130a regulates endothelial cell functions via the reactive oxygen species-activated NF-kB signaling pathway to promote skeletal muscle angiogenesis in diseases characterized by capillary rarefaction or inadequate angiogenesis (Lou et al., 2021). Moreover, Wang et al. and Ma et al. focused on how moderate exercise-induced exosomal miR-126 promotes angiogenesis and vascular repair by activating cardiovascular-related signaling pathways to inhibit endothelial cell apoptosis (Ma et al., 2018; Wang et al., 2020).

Beides, exercise-induced exosomes improve cardiovascular function by inhibiting myocardial fibrosis. Chaturvedi et al. showed that exercise-induced exosomes from cardiomyocytes (cardiosomes) miR-29b and miR-455-1 downregulate the expression of matrix metalloprotein (MMP9) to inhibit myocardial fibrosis and myocyte uncoupling (Chaturvedi et al., 2015). Moreover, suppressing exosomal iNOS derived from brown adipose tissue (BAT) can alleviate the expression of fibrotic genes in cardiac fibroblasts and reduce cardiac fibroblast dysfunction to reverse exosome-aggravated cardiac remodeling (Lin et al., 2022). A large number of experiments have shown that cardiomyocyte-derived exosomes may be superior to other cells and can play a protective role in cells, and the enhancement of protective factors can improve their ability (Kang et al., 2018). In addition, exercise-derived exosomal microRNAs are potent regulators of cardiomyocyte survival and functional properties, cardiomyocyte progenitor cells, and endothelial cells (Wu et al., 2021). Exosome miR-455-3p derived from bone marrow mesenchymal stem cells (BMMSCs) also has cardioprotective effects, targeting the MEKK1-MKK4-JNK signaling pathway to prevent myocardial I/R injury (Wang and Shen, 2022). Furthermore, using miRNA deep sequencing and RT-qPCR verification, we found that some known miRNAs were differentially expressed in circulating exosomes derived from the treadmill running model. In this bioinformatic study, we found that exosomal-miR-138-5p and EV-miR-10b-5p suppress inflammation and target MAPK and Caspase 9 to promote angiogenesis against MI/R (Oliveira et al., 2018; Huang et al., 2021).

Finally, It is an important advantage of exercise-derived exosomes as CVD biomarkers is that they can travel in blood and other fluids to reach endothelial cells, vascular smooth muscle cell, myocardial cell, and heart, skeletal muscle and other tissue/organs, and which are implicated in several pathophysiological processes throughout CVD development. In particular exosome-containing miRNAs have been suggested as biomarkers for the diagnosis and prognosis of MI/R and endothelial dysfunction, such as this rise in miR-138-5p, miR-10b-5p and miR-103-3p wascorrelated to angiogenesis and myocardial apoptosiss (Oliveira et al., 2018; Huang et al., 2021). Exosomal-miR-29b and miR-455-1 expression were positively associated with the myocardial fibrosis and myocyte uncoupling (Chaturvedi et al., 2015). In line with this, exosomes seem to play a role in cardiac fibrosis following myocardial in farction, and accordingly may be biomarkers in early diagnosis of CVD (Zara et al., 2020).

In summary, research shows that the miRNAs and proteins of exercise-induced exosomes released from various cells regulate the expression of target genes and play essential roles in inhibiting myocardial apoptosis, alleviating myocardial fibrosis, promoting angiogenesis and postinfarction myocardial repair, and increasing microvessel density to protect endothelial cells via intercellular communication. However, this review is especially noteworthy in exploring the evidence of the potential therapeutic effect of exercise-derived exosomes in the mechanisms of CVD. This indicates that exercise-derived exosomes and miRNAs can be used as biomarkers for the diagnosis and prognosis of CVD. In addition, these findings provide new mechanistic insights into the effects of exercise on cardioprotection and revascularization. miR-125b-5p, miR-122-5p, miR-342-5p, miR-126, miR-130a, miR-138-5p, and miR-455 were identified as novel miRNAs that mainly regulate the MAPK, NF-kB, VEGF, and Caspase signaling pathways, highlighting their potential therapeutic role in the prevention and treatment of CVD. Therefore, exosomal miRNAs, as specific target molecules involved in various signaling pathways, provide new therapeutic options for MI/R, myocardial infarction, heart failure, and cardiomyopathy. The gold standard for evaluating CVD must be efficient, but further clinical trials are needed to confirm the effects of exosomes for targeted interventional therapy.

### Limitation

The limitations of this systematic review and meta-analysis should also be noted. First, some data could not be converted to the SMD, which limited the inclusion of all available data in our meta-analysis. Second, all studies only enrolled male participants. The different populations may lead to biases and further affect the experimental results. Third, including experimental subjects is not uniform for human, animal, or cellular experiments, thus producing a significant bias. Fourth, we focused on the changes between the treatment (pre) and control (post) interventions; thus, the bias may have been reduced. Fifth, there was a lack of unanimous isolation, characterization, and application techniques, and we were not stringent in our inclusion and exclusion criteria.

## Conclusion

The present review summarizes different types of physical exercise that release exosomes into circulation and modify both protein and nucleic acid content, especially miRNAs, which play essential roles in protecting against CVD. However, further investigation is warranted to examine the effects of exercise-induced exosome release and its cargo in various conditions and CVD for potential therapeutic applications.
