# Corrigendum: Exosomes as a delivery tool of exercise-induced beneficial factors for the prevention and treatment of cardiovascular disease: a systematic review and meta-analysis

**DOI:** 10.3389/fphys.2024.1371224

**Published:** 2024-03-20

**Authors:** Zhijie Lai, Jiling Liang, Jingfeng Zhang, Yuheng Mao, Xinguang Zheng, Xiang Shen, Wentao Lin, Guoqin Xu

**Affiliations:** ^1^ Department of School of Physical Education, Guangzhou College of Commerce, Guangzhou, China; ^2^ College of Sports Medicine, Wuhan Sports University, Wuhan, China; ^3^ College of Humanities Education, Foshan University, Foshan, China; ^4^ Department of Sports and Health, Guangzhou Sport University, Guangzhou, China; ^5^ Department of School of Physical Education, Zhuhai College of Science and Techology, Zhuhai, China; ^6^ Guangdong Provincial Key Laboratory of Physical Activity and Health Promotion, Guangzhou Sport University, Guangzhou, China

**Keywords:** exercise, exosomes, extracellular vesicles, cardiovascular disease, meta-analysis

In the published article, there was an error in the **Author List**. The symbol (†) to indicate equal contribution and shared first authorship was missed from the author Zhijie Lai.

The authors apologize for this error and state that this does not change the scientific conclusions of the article in any way. The original article has been updated.

